# Integrating Single-Cell Profiling with Generative AI for *De Novo* Design of MMP9 Protein Binders in Diffuse Large B-Cell Lymphoma

**DOI:** 10.3390/molecules31111969

**Published:** 2026-06-05

**Authors:** Ziyang Miao, Siyi Zhu, Liwei Qin, Dawei Ma, Mingyang Lai, Pingping Xu, Yaping Jin, Huimin Cai, Shuai Zhao, Yang Wang

**Affiliations:** Hubei Key Laboratory of Industrial Biotechnology, College of Life Sciences, Hubei University, Wuhan 430062, China; 202331107010071@stu.hubu.edu.cn (Z.M.); 202431107010026@stu.hubu.edu.cn (S.Z.); 202421107011600@stu.hubu.edu.cn (L.Q.); 202331107010068@stu.hubu.edu.cn (D.M.); 202331107010101@stu.hubu.edu.cn (M.L.); xupingping@stu.hubu.edu.cn (P.X.); 202331107010015@stu.hubu.edu.cn (Y.J.); 202331107010024@stu.hubu.edu.cn (H.C.)

**Keywords:** MMP9, DLBCL, *de novo* protein design, single-cell transcriptomics, generative AI, protein-protein interaction

## Abstract

To clarify the cellular origin of matrix metalloproteinase-9 (MMP9) and explore targeted research, we utilized single-cell RNA sequencing analysis, which revealed that MMP9 is predominantly enriched in specific macrophages within the activated B-cell-like (ABC) subtype. Guided by this target information, we applied a generative AI pipeline incorporating RFdiffusion, ProteinMPNN, and AlphaFold to *de novo* design protein binders targeting the hemopexin (PEX) domain of MMP9. ELISA experiments confirmed the in vitro binding capability of these designs; among them, MMP9-30 displayed the strongest binding, with an apparent EC50 of approximately 1.1 μM, followed by MMP9-34, while MMP9-97 showed the weakest interaction. This study successfully integrates single-cell sequencing with AI-assisted protein design, providing a preliminary exploratory framework for subsequent MMP9-targeted research and protein binder development.

## 1. Introduction

Matrix metalloproteinase 9 (MMP9) is a zinc-dependent endopeptidase that plays a central role in extracellular matrix (ECM) remodeling, inflammatory signaling, and immune cell migration. Dysregulated MMP9 activity is associated with various pathological conditions, including chronic inflammation, autoimmune diseases, and cancer progression. In hematologic malignancies, such as diffuse large B-cell lymphoma (DLBCL), MMP9 promotes tumor invasion and immune microenvironment remodeling by degrading ECM components and modulating the availability of cytokines [1,2].

Despite rapid advances in both single-cell transcriptomics and computational protein design, these two fields have rarely been integrated into a unified discovery framework [3,4,5]. Single-cell analysis enables the identification of disease-relevant molecular targets with cell-type resolution [4,6,7], whereas generative protein design technologies provide a powerful strategy for engineering novel binders against structurally defined targets. However, most protein design studies still rely on predefined targets rather than data-driven target discovery [1,8,9].

**Figure 1:**
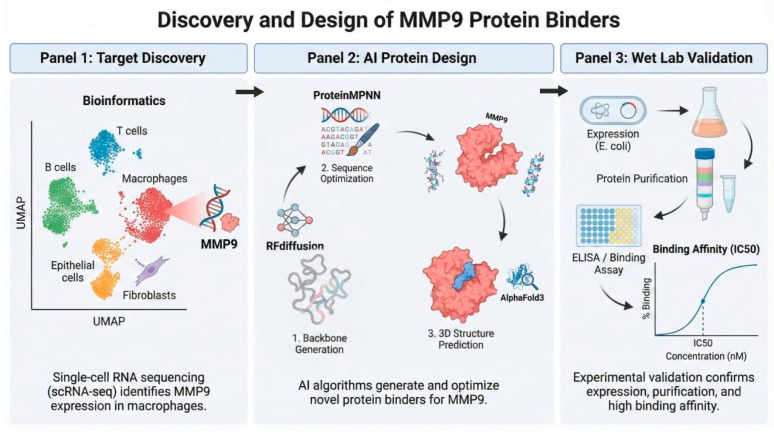
The discovery and design of MMP9 protein binders: target discovery, AI-assisted protein design, and wet-lab validation.

In this study, we combined single-cell transcriptomic analysis with generative AI-based protein design to achieve precise localization and explore targeted strategies against MMP9 in DLBCL (Figure 1). Using diffuse large B-cell lymphoma as a model, we first characterized the specific expression profile of MMP9 in macrophage subpopulations via single-cell analysis. Traditionally, targeting MMPs has been challenging due to the high structural conservation of their catalytic domains, which frequently leads to off-target effects. To address this, we applied a diffusion-based computational pipeline to *de novo* design protein binders specifically targeting the unique hemopexin (PEX) domain of MMP9. Selecting the PEX domain serves as an inherent negative design principle to circumvent high sequence homology and ensure target specificity [10,11,12]. This study demonstrates how integrating multi-omics data with computational structural biology can provide a preliminary exploratory framework for key molecules within complex microenvironments.

## 2. Results

### 2.1. Single-Cell Transcriptomic Analysis Identifies MMP9 as a Macrophage-Enriched Target in ABC-Type DLBCL

**Figure 2:**
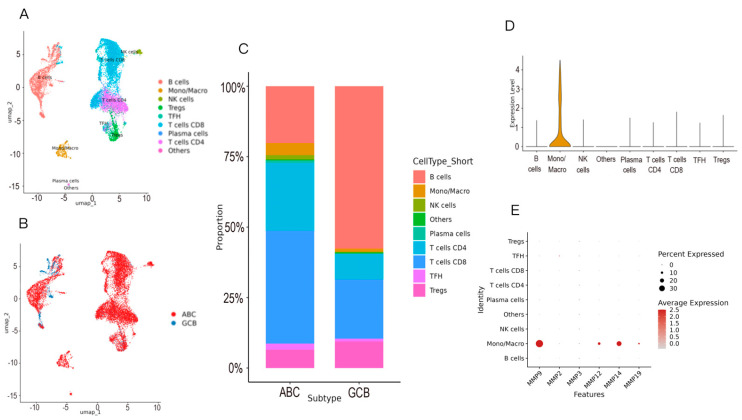
Bioinformatic Analysis: MMP9 is Highly Expressed in the Monocyte/Macrophage Population. (**A**) UMAP visualization colored by cell type. (**B**) UMAP visualization colored by molecular subtype. (**C**) Proportional composition of cell types across subtypes. (**D**) MMP9 expression across cell types (highly expressed in monocytes/macrophages). (**E**) Expression profile of MMP family genes across cell types.

To delineate the cellular origin and subtype-specific expression pattern of Matrix metalloproteinase-9 (MMP9) in diffuse large B-cell lymphoma (DLBCL), we analyzed single-cell RNA sequencing (scRNA-seq) data encompassing both activated B-cell-like (ABC) and germinal center B-cell-like (GCB) subtypes. Unsupervised clustering combined with UMAP dimensionality reduction resolved the tumor microenvironment into major cellular compartments, including malignant B cells, T cell subsets (CD4^+^, CD8^+^, Treg, Tfh), natural killer (NK) cells, plasma cells, and monocytes/macrophages (Mono/Macro) (Figure 2A).

Comparative analysis of cellular composition revealed a marked enrichment of monocytes/macrophages in ABC-DLBCL relative to the GCB subtype (Figure 2C). Examination of MMP9 transcript distribution demonstrated that MMP9 expression was highly restricted to the monocyte/macrophage compartment (Figure 2B). The majority of MMP9-expressing cells were localized within the monocyte/macrophage compartment, whereas malignant B cells and other immune populations exhibited near-baseline expression levels. Consistent with this observation, MMP9 expression was markedly enriched in monocytes/macrophages compared with other cell types. In contrast, other MMP family members, including MMP2, MMP3, MMP12, and MMP14, did not display comparable cell-type specificity (Figure 2D), indicating a distinctive expression pattern for MMP9 within the tumor microenvironment.

**Figure 3:**
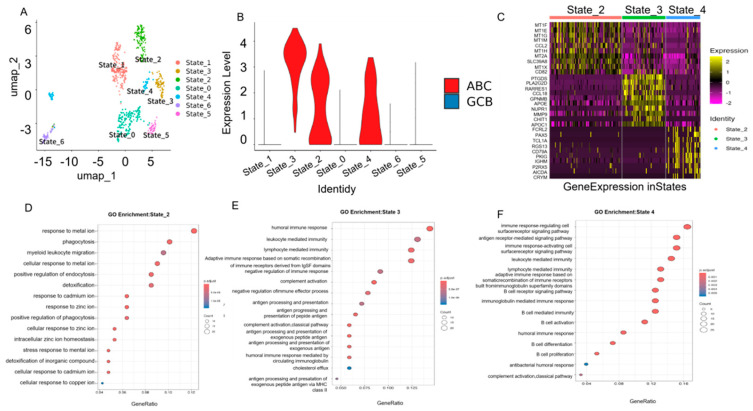
Bioinformatic Analysis: Seven States within the Monocyte/Macrophage Population. (**A**) UMAP visualization colored by the seven transcriptionally distinct states. (**B**) MMP9 expression levels across the seven states. States 2, 3, and 4 highly express MMP9. (**C**) Signature genes for the three MMP9-high states (State_2, State_3, State_4). (**D**,**E**,**F**) Gene Ontology (GO) enrichment results for each respective state.

To further characterize macrophage heterogeneity, we performed sub-clustering analysis of the monocyte/macrophage population, identifying seven transcriptionally distinct macrophage states (State_0 to State_6) (Figure 3A). Quantitative comparison revealed that MMP9 expression was concentrated in three states—State_2, State_3, and State_4—particularly within the ABC-DLBCL samples (Figure 3B). These three states accounted for the majority of MMP9-high macrophages, while the remaining four states exhibited minimal or undetectable expression.

Marker gene analysis revealed distinct transcriptional programs among the MMP9-high states (Figure 3C). State_2 was characterized by elevated expression of metal ion–responsive and inflammatory genes, including MT1 family members (MT1F, MT1E, MT1G), CCL2, GPNMB, and APOE. State_3 showed enrichment of genes associated with extracellular matrix remodeling and immune regulation, such as MMP9, CHIT1, and APOC1. State_4 expressed genes involved in humoral immune processes and immune cell interaction, including FCRL2, PAX5, IGHM, and AICDA. Despite the presence of B cell–associated transcripts in State_4, these cells retained canonical macrophage markers (e.g., CSF1R, CD68), supporting their classification as macrophage-derived rather than technical doublets.

Gene Ontology (GO) enrichment analysis further supported functional distinctions among these states. State_2 was enriched for biological processes related to metal ion response, phagocytosis, and detoxification of inorganic compounds (Figure 3D); State_3 was associated with immune regulation, complement activation, and antigen processing and presentation (Figure 3E); and State_4 showed enrichment in pathways linked to humoral immune response and B cell receptor signaling (Figure 3F).

Collectively, these results demonstrate that MMP9 expression in DLBCL is predominantly confined to specific macrophage functional states and is more pronounced in the ABC subtype, establishing a cell-type-resolved expression landscape for MMP9 within the tumor microenvironment.

### 2.2. De Novo Design of MMP9 Protein Binders Using a Generative AI Pipeline

Based on the cell-type–resolved identification of MMP9 as a macrophage-associated target, we next explored whether generative AI–based protein design could be used to generate candidate binders against this protein. The hemopexin (PEX) domain of MMP9 (PDB ID: 1ITV) was selected as the structural target. Specifically, targeting this unique PEX domain rather than the highly conserved catalytic domain served as an inherent negative design strategy to ensure high specificity and prevent off-target binding to other MMP family members [10,11,12]. Subsequently, RFdiffusion was employed to generate binder backbones with geometric complementarity to the target surface.

Under minimal geometric constraints and without predefined binding motifs, RFdiffusion sampled a diverse set of backbone conformations positioned at the MMP9 surface. The resulting candidate binders were approximately 50 amino acids in length and displayed heterogeneous backbone topologies. These backbones were subsequently subjected to sequence optimization using ProteinMPNN, with solubility-biased model weights applied to favor amino acid compositions compatible with stable folding and recombinant expression.

Initial structural evaluation was performed using AlphaFold-Multimer (AlphaFold2) to assess the complex formation between the designed binders and MMP9. High-confidence candidate complexes were then subjected to AlphaFold3 for deep interface refinement. Designs were filtered based on multiple structural quality metrics, including predicted Local Distance Difference Test (pLDDT), Predicted Aligned Error (PAE) at the binding interface, and binder backbone RMSD relative to the designed conformation. This multi-stage screening process yielded a subset of candidates exhibiting stable predicted folding and well-defined binding interfaces.

**Figure 4:**
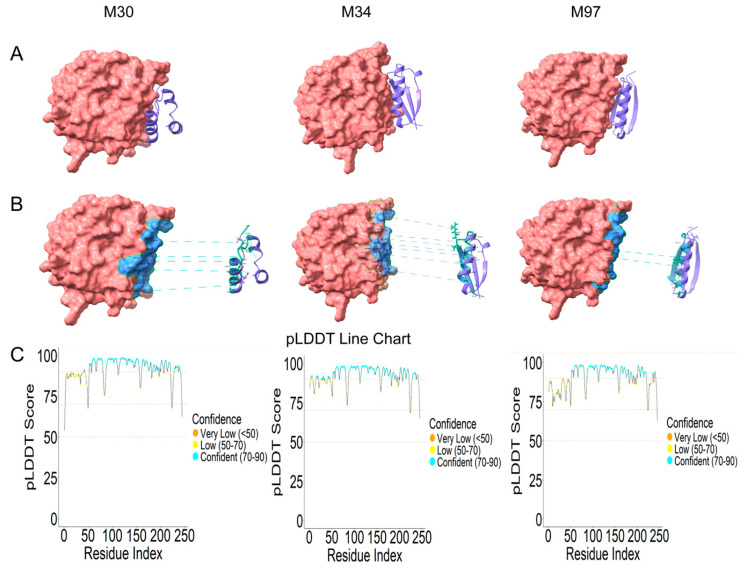
Protein design results. (**A**) Schematic representation of MMP9 in complex with the corresponding designed binders. (**B**) Illustration of the binding interfaces between MMP9 and the corresponding binders. (**C**) pLDDT profiles of the corresponding binders, used to demonstrate the quality and confidence of the designs.

Three representative designs—designated M30, M34, and M97—were selected for further analysis based on interface geometry and structural diversity. Predicted complex structures generated by AlphaFold2 showed that all three binders associate with the MMP9 surface in a consistent orientation (Figure 4A). The binders occupy surface grooves of the PEX domain and form continuous interfaces without apparent steric clashes.

Furthermore, the robustness of the designed binders was supported by multiple structure confidence metrics derived from AlphaFold2 and AlphaFold3 predictions. High predicted Local Distance Difference Test (pLDDT) scores across the binder structures indicate reliable folding, while low Predicted Aligned Error (PAE) values at the binding interfaces suggest well-defined and confident inter-chain positioning. Together, these metrics provide strong computational evidence for the structural stability and reliability of the designed protein–protein complexes.

Interface analysis of the predicted complex structures, performed using UCSC ChimeraX, revealed that each binder establishes multiple intermolecular contacts with MMP9, including hydrogen bonds and hydrophobic interactions identified based on standard geometric criteria (Figure 4B). Furthermore, the selected binders exhibited consistently high predicted Local Distance Difference Test (pLDDT) scores at the binding interfaces, supporting the structural credibility of the modeled complexes. Although the overall binding modes were similar, the spatial distribution and density of interface residues differed among the three designs, suggesting variability in interfacial complementarity.

Predicted pLDDT profiles indicated high structural confidence across most residues of the designed binders (Figure 4C). The mean pLDDT values for M30, M34, and M97 were 91.59, 92.22, and 90.77, respectively, with localized reductions primarily observed at terminal regions. Interface regions maintained consistently high confidence scores, supporting the structural stability of the predicted complexes.

Together, these results demonstrate that the generative design pipeline produced structurally well-folded protein binders with defined binding interfaces to MMP9, providing a set of candidates suitable for experimental validation. 

### 2.3. In Vitro Experimental Application and Quantitative Binding Assessment of MMP9 Binders

**Figure 5:**
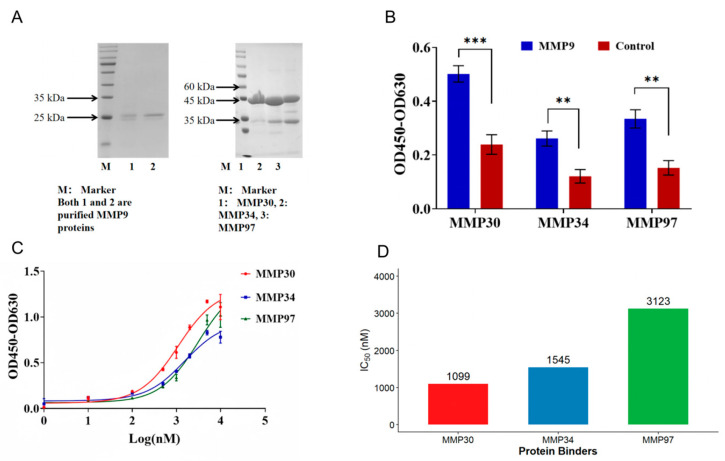
Experimental Validation. (**A**) Protein Purification Verification. Successful protein expression and purification were confirmed. (**B**) Single-Point ELISA Specific Binding Test. The binding signals of all three designed proteins to MMP-9 were significantly higher than those of the control group, demonstrating strong binding specificity. (**C**) Dose–Response Curve. (**D**) Apparent EC50 values derived from ELISA-based dose–response assays. MMP30 emerged as the best-binding protein among the three candidates (apparent EC50 ≈ 1.1 μM), followed by MMP34, with MMP97 being the weakest. All quantitative data are presented as the mean ± standard deviation (SD) of three independent biological replicates (*n* = 3). Statistical significance in (**B**) was determined using a Student’s *t*-test (**, *p* < 0.01; ***, *p* < 0.001). The dose–response curves in (**C**) and the apparent EC50 values in (**D**) were calculated using a non-linear regression four-parameter logistic (4PL) model.

To experimentally evaluate the binding performance of the designed proteins, three representative candidates—designated MMP9-30, MMP9-34, and MMP9-97—were selected based on predicted interface quality and structural diversity. Recombinant MMP9 and the designed binders were successfully expressed and purified, as confirmed by SDS–PAGE analysis, which showed bands consistent with the expected molecular weights and high purity (Figure 5A).

Specific binding of the designed proteins to MMP9 was first assessed using an enzyme-linked immunosorbent assay (ELISA). At a fixed binder concentration, all three candidates produced significantly higher binding signals to immobilized MMP9 compared with negative controls, indicating specific target recognition (Figure 5B).

To further quantify binding behavior, ELISA-based dose–response experiments were performed using serial dilutions of each binder. All three candidates exhibited concentration-dependent binding curves characteristic of protein–protein interactions (Figure 5C). Apparent binding constants were estimated by fitting the dose–response curves, revealing micromolar-range affinities for all three binders. Among them, MMP9-30 displayed the strongest binding, with an apparent EC50 of approximately 1.1 μM, followed by MMP9-34, while MMP9-97 showed the weakest interaction (Figure 5D).

Although the designed binders demonstrated clear specificity toward MMP9, none of the candidates achieved sub-micromolar apparent affinity under the tested conditions. These results indicate that the first-generation binders are capable of engaging MMP9 but exhibit moderate binding strength in vitro.

To provide orthogonal biophysical validation of the interactions, Bio-Layer Interferometry (BLI) was additionally performed. Consistent with the ELISA observations, the BLI sensorgrams confirmed the specific, concentration-dependent binding of the variants to the MMP9 PEX domain. The calculated equilibrium dissociation constants (KD) were in the low micromolar range (M30: 2.86 ± 0.09 μM; M34: 4.52 ± 0.23 μM; M97: 10.75 ± 0.20 μM). Detailed kinetic profiles and sensorgrams are provided in the Supplementary Materials (Figure S4). These orthogonal measurements further substantiate the validated functional scaffolds generated by our computational workflow.

## 3. Discussion

In this study, we combined single-cell transcriptomic analysis with generative AI-driven protein design to achieve precise localization and targeted intervention of matrix metalloproteinase 9 (MMP9) in diffuse large B-cell lymphoma (DLBCL). By resolving the cellular source of MMP9 expression at single-cell resolution and subsequently designing de novo protein binders against this target, our work demonstrates the effective integration of multi-omics profiling with structure-based molecular engineering.

### 3.1. Single-Cell Transcriptomics Reveals Macrophage-Associated MMP9 Expression in DLBCL

Single-cell transcriptomic analysis provides an increasingly powerful approach for dissecting the cellular heterogeneity of tumor microenvironments [4,13,14]. In diffuse large B-cell lymphoma (DLBCL), the tumor microenvironment is composed of multiple immune and stromal cell populations that collectively influence disease progression, therapeutic response, and immune regulation. Among these components, tumor-associated macrophages have been implicated in extracellular matrix remodeling, immune modulation, and lymphoma progression.

In the present study, single-cell RNA-sequencing analysis revealed that MMP9 expression is predominantly enriched in macrophage-associated cell states within ABC-type DLBCL. This observation is consistent with previous reports suggesting that macrophages represent an important source of matrix metalloproteinases in tumor microenvironments [15,16,17,18]. Because MMP9 is a key regulator of extracellular matrix degradation and tissue remodeling, macrophage-derived MMP9 may contribute to tumor invasion, immune cell recruitment, and microenvironmental remodeling.

Rather than serving solely as a descriptive observation, the identification of macrophage-associated MMP9 expression provided a biologically informed entry point for downstream target-driven molecular design. In this context, single-cell transcriptomics offers an advantage over bulk transcriptomic approaches by enabling the prioritization of targets within specific cellular compartments of the tumor microenvironment [18,19,20].

### 3.2. Generative AI-Assisted Design of Protein Binders Targeting MMP9

Recent advances in generative artificial intelligence have significantly expanded the possibilities for *de novo* protein engineering. Diffusion-based protein design methods, combined with sequence optimization algorithms and structure prediction tools, now allow the rapid generation of candidate protein binders with defined structural constraints [5,21,22,23,24,25].

In this study, we implemented a generative design pipeline integrating RFdiffusion, ProteinMPNN, and AlphaFold-based structural evaluation to design compact protein binders targeting the hemopexin (PEX) domain of MMP9. This workflow enables the automated generation of backbone structures, sequence optimization for structural stability, and structural validation of predicted protein–protein interactions.

Experimental validation demonstrated that representative designed proteins can bind MMP9 in ELISA-based binding assays, with the best candidate exhibiting an apparent EC50 in the micromolar range. Although these affinities remain modest, they indicate that the computational pipeline is capable of generating proteins that recognize the target under in vitro conditions. Because our workflow performed a global design across the PEX domain, we hypothesize that this moderate affinity partially stems from steric competition with MMP9’s robust native homodimerization process. Similar affinity ranges have frequently been reported for first-generation computationally designed binders prior to affinity maturation, suggesting that further optimization—such as site-directed disruption of the dimer interface—could improve binding strength [5,22,26].

Importantly, the goal of the present study was not to produce fully optimized therapeutic inhibitors but rather to demonstrate the feasibility of integrating single-cell target discovery with generative protein design as a unified discovery framework.

### 3.3. Structural Constraints, Global Design, and Binding Affinity

While the hemopexin (PEX) domain of MMP9 was selected as the structural target for binder design, it is important to distinguish between biological target identification and structural interface selection. In this study, single-cell transcriptomic analysis was primarily used to identify MMP9 as a biologically relevant molecule enriched in specific macrophage states within the DLBCL microenvironment. The choice of the PEX domain for computational binder design was instead guided by structural considerations, particularly as a core negative design principle. Because the catalytic domains of MMP family members share high sequence and structural homology, targeting the catalytic pocket often leads to severe off-target binding. By specifically targeting the unique PEX domain, we inherently introduced a negative design constraint to circumvent cross-reactivity and achieve high binding specificity.

It is noteworthy that the design strategy employed in this study falls within the global design paradigm—namely, exploring potential binding configurations across the entire PEX domain through expansive conformational space searches in the absence of a predefined target pocket. While this approach facilitates the discovery of novel binding interfaces at an early stage, it inherently offers limited capacity for fine-grained optimization of local interfaces. Crucially, MMP9 possesses a complex structural architecture and a strong natural propensity to undergo homodimerization mediated precisely by its PEX domain [26,27,28].

Because our computational workflow performed an unbiased global sampling without specifically avoiding or intentionally disrupting this dimer interface, the resulting binders likely experience severe steric hindrance or direct competition with MMP9’s robust native homodimerization process in solution. These structural dynamics provide a compelling and plausible explanation for the moderate, micromolar-range affinities observed for the first-generation binders reported in this study. In other words, global design prioritized the identification of feasible specific binding modes over the direct attainment of high-affinity solutions capable of outcompeting native dimerization.

The precise structural determinants governing binding affinity remain to be fully characterized, and the influence of PEX oligomerization warrants further investigation using high-resolution experimental techniques. Furthermore, since MMP9 homodimerization has been reported to accelerate extracellular matrix degradation, disrupting this specific interaction presents a highly promising therapeutic avenue. Taken together, while the global targeting of the PEX domain represents a structurally tractable site for initial binder discovery, future optimization strategies should explicitly shift towards site-directed computational design targeting the critical residues mediating MMP9 homodimerization, in addition to employing affinity maturation and multivalent design approaches to achieve robust functional modulation [3,28].

Beyond structural optimization, translating these de novo binders into effective interventions necessitates integrating them with the microenvironmental context identified in our single-cell analysis. Because our transcriptomic data specifically localized MMP9 enrichment to macrophage-associated niches within the DLBCL microenvironment, our upcoming in vivo models will focus on directing these peptides to these specific sites. Employing advanced delivery platforms, such as targeted nanomaterials or peptide-drug conjugates, will be crucial to protect the binders from proteolytic degradation and ensure their localized accumulation. Ultimately, pairing these rationally designed binders with microenvironment-targeted delivery systems will provide a cohesive translational pipeline, effectively coupling our initial bioinformatic target discovery with localized in vivo exploratory research.

### 3.4. Biological Implications and Functional Limitations of the Designed Binders

An important question concerns the potential biological consequences of binder interaction with MMP9. Because the designed proteins target the hemopexin (PEX) domain of the enzyme, their primary mechanism of action would not be direct active-site inhibition. Instead, such interactions may sterically hinder PEX-mediated homodimerization or alter conformational dynamics. Given that MMP9 dimerization accelerates extracellular matrix degradation and facilitates protein–protein interactions essential for cell migration, the designed binders could allosterically or sterically interfere with these critical functions.

In the context of diffuse large B-cell lymphoma, MMP9 has been implicated in extracellular matrix remodeling, immune microenvironment regulation, and tumor invasion. Consequently, potential steric interference with MMP9’s functional protein–protein interactions by the designed binders could influence downstream processes such as extracellular matrix degradation, immune cell migration, or tumor-associated stromal interactions [3,9].

However, the present study focuses on demonstrating the feasibility of this computational design strategy as an initial conceptual validation. While computational metrics (such as high AlphaFold2 pLDDT scores) and the robust solubility of the expressed proteins strongly support their structural integrity, we recognize that circular dichroism (CD) spectroscopy or NMR would provide definitive experimental evidence of solution conformations for subsequent affinity optimization. Furthermore, while specific in vitro binding was confirmed, extensive functional assays evaluating enzymatic inhibition, extracellular matrix degradation, or DLBCL cell invasion remain beyond the scope of this current work.

Future in vitro and in vivo studies will therefore be required to determine the precise extent to which these PEX-targeted binders influence MMP9-driven microenvironmental remodeling and lymphoma progression, which would provide further insight into their potential biological and translational relevance.

## 4. Materials and Methods

### 4.1. Data Sources and Analytical Methods

#### 4.1.1. Single-Cell Transcriptomic Data Processing and Cell Atlas Construction

The raw gene expression matrix and cell metadata for dataset GSE182434 were obtained from the GEO database [14]. Data analysis was performed using the Seurat package (v5.3.0, New York Genome Center, New York, NY, USA) [29]. Samples FL1, FL2, FL3, and T2 were excluded from the analysis. Quality control was strictly applied to remove low-quality cells and technical artifacts; cells were retained if they exhibited a mitochondrial gene percentage < 15% and a detected gene count between 200 and 6000. The filtered expression data were normalized using the NormalizeData function, and the top 2000 highly variable genes were identified via variance-stabilizing transformation (vst) [29]. Batch effects were corrected using Harmony based on sample identity. Dimensionality reduction via UMAP and cell clustering were performed based on the first 30 Harmony principal components [30]. Cell types were annotated manually according to canonical marker genes (e.g., B cells: MS4A1, CD79A; Macrophages: CSF1R, CD68; T cells: CD3D, CD3E). Cells were further classified into ABC and GCB molecular subtypes based on the metadata, with proportional composition visualized using the ggplot2 package.

#### 4.1.2. Gene Expression Feature Analysis

The expression distribution of MMP9 across cell types was visualized using violin plots (VlnPlot). To compare expression patterns within the MMP family, a core set of members (MMP2, MMP3, MMP9, MMP12, MMP14, MMP19) was selected, and their expression profiles were visualized using dot plots (DotPlot), where color intensity represents the average scaled expression level and dot size indicates the percentage of expressing cells [29].

#### 4.1.3. Macrophage Subpopulation Re-Clustering Analysis

The monocyte/macrophage subpopulation was extracted by subsetting cells annotated as “Macro” or “Mono”. An independent Seurat object was constructed for this subset. Following normalization, variable feature identification, and scaling, principal component analysis (PCA) was performed. Batch effects were corrected using Harmony [30]. UMAP dimensionality reduction (dims = 1:20) and graph-based clustering (resolution = 0.3) yielded seven transcriptionally distinct macrophage states (State_0 to State_6). MMP9 expression across these states was visualized, identifying State_2, State_3, and State_4 as the primary MMP9-expressing subpopulations [29].

#### 4.1.4. Functional Annotation of Macrophage States

Differential gene expression analysis was performed specifically for the MMP9-high states (State_2, State_3, State_4) using a “one vs. rest” strategy. Significantly upregulated marker genes for each state were identified using the Wilcoxon rank-sum test (adjusted *p*-value < 0.05, average log2FC > 0.25). The top 10 marker genes for each state were visualized in a heatmap. For functional annotation, marker genes were mapped to Entrez IDs using the clusterProfiler package, and Gene Ontology (GO) Biological Process enrichment analysis was conducted (pAdjustMethod = “fdr”, pvalueCutoff = 0.05) [31]. The top 15 most significantly enriched GO terms for each state were visualized using dot plots.

### 4.2. Design of MMP9 Binders

#### 4.2.1. Overview of Computational Tools

The overall computational workflow for generating and optimizing MMP9-targeting binders proceeded as follows: (1) *de novo* backbone generation complementary to the target structure using RFdiffusion (v1.1.0, University of Washington, Seattle, WA, USA) [5]; (2) sequence design on fixed backbones using ProteinMPNN (v1.0.0, University of Washington, Seattle, WA, USA) [22]; (3) initial complex pre-screening using AlphaFold-Multimer(AlphaFold2) (v2.3.0, Google DeepMind, London, UK) [23]; (4) complex interface refinement and high-resolution assessment using AlphaFold3 (Web Server, Google DeepMind, London, UK) [24].

#### 4.2.2. MMP9 Binder Backbone Generation

Binder backbones were generated using the standard mode of RFdiffusion. The crystal structure of the MMP9 hemopexin (PEX) domain (PDB ID: 1ITV [27]) served as the target. During inference, the generation region was constrained using the contigmap protocol to target chain B (residues 1–195) and generate a binder of 50 residues (configuration: [‘B1-195/0 50-50’]). Under this configuration, the model generated binder conformations spatially complementary to the MMP9 PEX domain surface *de novo*, without reliance on external scaffold templates. A focused library of backbone candidates was generated for subsequent processing.

#### 4.2.3. Design Funnel and Candidate Selection

To improve transparency and reproducibility of the computational workflow, the design pipeline followed a multi-stage screening strategy. RFdiffusion was first used to generate approximately 500 backbone candidates positioned on the surface of the MMP9 hemopexin (PEX) domain. These backbones were generated without predefined binding motifs to allow diverse structural sampling of potential interaction geometries.

For each backbone, 8–16 sequence variants were generated using ProteinMPNN, resulting in several thousand candidate designs. Sequence design was performed using the soluble protein model weights to favor amino-acid compositions compatible with stable folding and soluble recombinant expression.

To strictly evaluate the computational robustness of these sequence designs, candidate binders were subsequently subjected to AlphaFold-based structure prediction as an in silico forward-folding validation. Filtering criteria were applied to select high-confidence designs, including:

- Mean predicted Local Distance Difference Test (pLDDT) > 85;
- Interface predicted aligned error (PAE) < 10 Å;
- Backbone RMSD relative to the designed conformation < 2 Å.

Designs satisfying these criteria were further inspected for interface complementarity and structural diversity. Based on these metrics, three representative candidates (M30, M34, and M97) were selected for experimental validation.

#### 4.2.4. Sequence Design

Following backbone generation, sequence design was performed using ProteinMPNN. To enhance physicochemical properties, we employed the pretrained soluble protein model weights (soluble_model_weights/v_48_002.pt). This setting promotes the enrichment of polar amino acids on the binder surface, thereby improving solubility in physiological environments and mitigating aggregation risks during heterologous expression.

#### 4.2.5. Structural Evaluation and Optimization

Initial binder-MMP9 complex formation was pre-screened using AlphaFold-Multimer (AlphaFold2). Subsequently, the AlphaFold3 server was utilized for deep refinement of the binder–MMP9 complex interface. Unlike its predecessor, AlphaFold3 incorporates a diffusion-based architecture that enables superior modeling of atomic-level details at protein–protein interfaces. During the screening process, candidates were filtered based on key structural quality metrics, including predicted Local Distance Difference Test (pLDDT), Predicted Aligned Error (PAE), and interface RMSD, to ensure high model stability and reliability.

### 4.3. In Vitro Experimental Validation

#### 4.3.1. Protein Expression and Purification

Plasmid Construction and Cloning The target gene fragments of MMP9 and the Binder were amplified using PCR (annealing temperature: 58 °C; extension time: 52 s), and the resulting products were identified via agarose gel electrophoresis. After digesting the template plasmids with the Dpn enzyme, the target fragments were recombined into the pET-28a vector (for MMP9) and the pET-23a vector (for the Binder) using T5 exonuclease. The recombinant products were transformed into competent cells. The MMP9 group was plated on LB solid medium containing kanamycin, while the Binder group was plated on LB solid medium containing ampicillin. Single colonies were subsequently selected and verified through colony PCR screening and DNA sequencing to ensure the sequences were entirely correct.

Protein Expression and Cell Lysis Sequencing-verified MMP9/pET-28a and Binder/pET-23a strains were inoculated into LB liquid media containing the corresponding antibiotics (kanamycin or ampicillin) and cultured at 37 °C with shaking until reaching the logarithmic growth phase. Protein expression was induced by the addition of IPTG and maintained at 18 °C for 18 h. The bacterial cells were harvested by centrifugation and resuspended in PBS buffer. Cell lysis was performed using a low-temperature ultra-high-pressure cell homogenizer at a pressure of 800–1000 bar. After high-speed refrigerated centrifugation of the lysate, the supernatants containing the target proteins were collected.

Protein Purification The supernatants were loaded onto pre-equilibrated Nickel affinity chromatography (Ni-NTA) columns. Non-specifically bound impurities were removed by washing with a low-concentration imidazole buffer, followed by the elution of the target proteins using a high-concentration imidazole buffer. The eluted fractions were collected, concentrated via ultrafiltration, and subjected to buffer exchange. Finally, fine purification was performed using size-exclusion chromatography (SEC) on an ÄKTA Pure system. The final target proteins were collected and stored.

#### 4.3.2. Binding Affinity Assay (ELISA)

The binding affinity between the proteins was determined using an Enzyme-Linked Immunosorbent Assay (ELISA) (Solarbio, Beijing, China). The MMP9 antigen was coated onto 96-well plates and incubated overnight at 4 °C. The following day, the coating solution was discarded, and the wells were washed three times with TBST buffer (5 min per wash) before being blocked with 100 μL of 1% BSA solution (Solarbio, Beijing, China) for 2 h. After blocking and another three washes, serially diluted samples (prepared in 1% BSA) were added and incubated for 1 h. Following three additional washes to remove unbound samples, 100 μL of 1% diluted HRP-conjugated mouse antiHA-Tag mAb (ABclonal, Wuhan, China) was added to each well and incubated for 1 h. After a final round of three washes, 100 μL of TMB substrate was added to each well. After a 30 min incubation, the absorbance was measured at OD450 and OD630 using a microplate reader. All binding and competitive inhibition assays were performed in three independent biological replicates (*n* = 3), and data are presented as mean ± standard deviation (SD). Statistical significance between groups was determined using a Student’s *t*-test. For dose–response curves, data were fitted using a non-linear regression four-parameter logistic (4PL) model to calculate the IC50 and apparent EC50 values.

#### 4.3.3. Biolayer Interferometry (BLI)

The streptavidin sensors were first equilibrated in PBS buffer at room temperature for 20 min to stabilize the sensor surface. Following equilibration, a stable baseline signal was recorded in PBS buffer for 60 s. The sensors were then transferred to a solution containing the biotinylated protein for 300 s to allow for immobilization. Subsequently, the sensors were washed in TBST buffer for 180 s to re-establish the baseline. The association phase was monitored by transferring the sensors to the analyte solution for 300 s, followed by a dissociation phase in TBST buffer for another 300 s to record the dissociation kinetics.

## 5. Conclusions

In summary, this study integrates single-cell transcriptomic analysis with generative AI-assisted protein design to identify MMP9 as a macrophage-associated target within the DLBCL microenvironment, and to generate a series of candidate binders targeting its hemopexin (PEX) domain through *de novo* design strategies. Although the first-generation binders exhibited binding affinities in the micromolar range, structural analysis indicates that this level of affinity is consistent with a global design phase, reflecting initial success in interface recognition despite inherent steric competition with the native MMP9 homodimerization process.

Future efforts will focus on several key directions. First, systematic enhancement of binding affinity will be pursued through computational affinity maturation and multivalent design strategies. Second, building upon our current negative design strategy of targeting the unique PEX domain to avoid conserved catalytic pockets, future iterations will explicitly shift towards site-directed computational design to precisely disrupt the critical residues mediating the MMP9 homodimerization interface. Finally, microenvironment-specific delivery systems—such as nanocarriers or peptide conjugation platforms—will be employed to achieve stable in vivo delivery and precise localization of the designed molecules.

Collectively, this study establishes an integrated framework spanning bioinformatics-based target discovery, structure-based design, and subsequent translational planning, providing a preliminary roadmap for the application of *de novo* protein design in targeted exploratory research within complex tumor microenvironments.

## Data Availability

The original contributions presented in the study are included in the article and its Supplementary Materials. Specifically, the designed protein sequences, molecular dynamics simulation data, experimental binding affinity (BLI) data, and 3D structural coordinates (PDB files) of the modeled complexes are provided within the Supplementary Materials. Any further inquiries can be directed to the corresponding author.

## Supplementary Materials

The following supporting information can be downloaded at: https://www.mdpi.com/article/10.3390/molecules31111969/s1, Figure S1: RMSD trajectory of the M30 binder in complex with MMP9; Figure S2: RMSD trajectory of the M34 binder in complex with MMP9; Figure S3: RMSD trajectory of the M97 binder in complex with MMP9; Figure S4: BLI sensorgrams for the binding of designed binders (M30, M34, M97) to the MMP9 PEX domain; Table S1: Amino acid sequences of the de novo designed MMP9 binders; Supplementary Data: 3D atomic coordinates of the computationally designed MMP9 binder complexes.
