# Supplementary material for: Integrating Single-Cell Profiling with Generative AI for De Novo Design of MMP9 Protein Binders in Diffuse Large B-Cell Lymphoma
Source: Molecules. 2026 Jun 5;31(11):1969. doi: 10.3390/molecules31111969 (PMC13257871; doi:10.3390/molecules31111969)
Supplement: Supplementary file 1 [file molecules-31-01969-s001.zip › Supplementary_Materials.pdf]

Supplementary Materials

Integrating Single-Cell Profiling with Generative AI for *De Novo* Design of MMP9 Protein Binders in Diffuse Large B-Cell Lymphoma

Ziyang Miao [1], Siyi Zhu [1], Liwei Qin [1], Dawei Ma [1], Mingyang Lai [1], Pingping Xu [1],Yaping Jin [1], Huimin Cai [1], Shuai Zhao [1*] and Yang Wang [1*]

1. Hubei Key Laboratory of Industrial Biotechnology, College of Life Sciences, Hubei University, Wuhan 430062, PR China

* Correspondence: yangwang@hubu.edu.cn; zhaoshuai@stu.hubu.edu.cn

Supplementary Figures

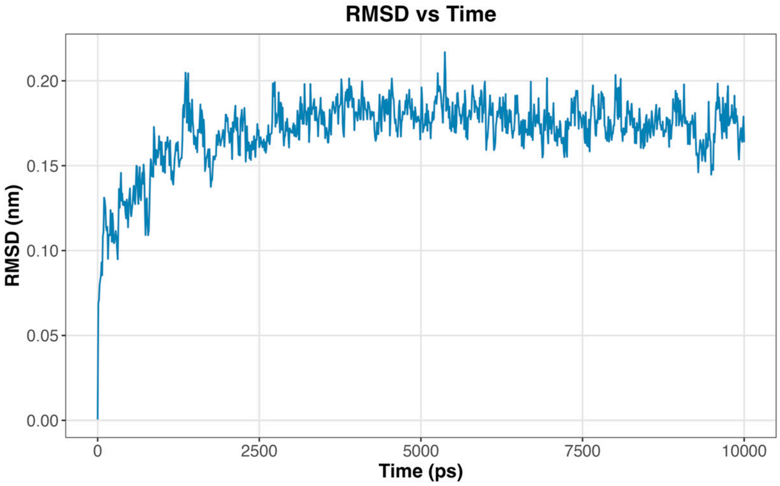

Figure S1

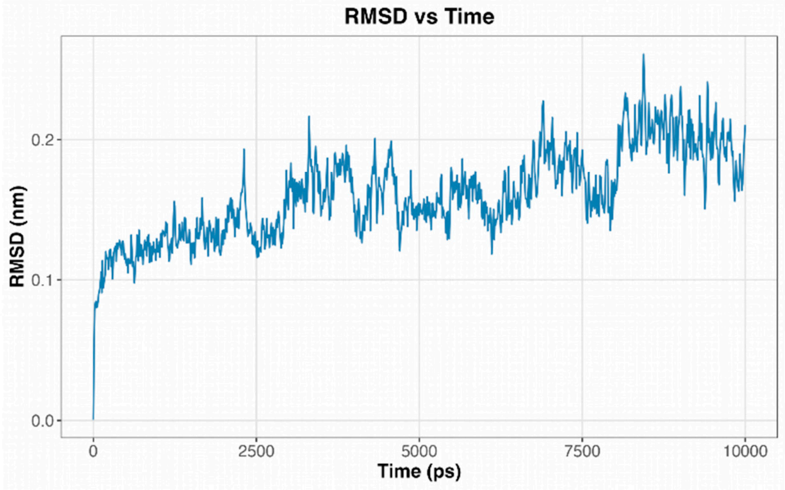

Figure S2

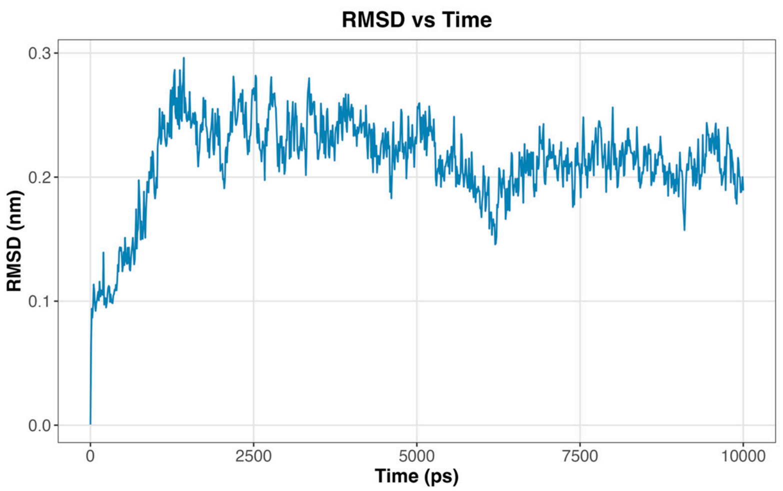

Figure S3

Figure S1–3 represent the RMSD trajectories of three independently designed binders in complex with MMP9. In all simulations, the RMSD increases during the initial equilibration stage and subsequently stabilizes, indicating structural relaxation of the complexes. In **Figure S1**, the RMSD stabilizes at ~0.16–0.19 nm after about 1 ns of simulation for the M30 binder. In **Figure S2**, the RMSD gradually rises from ~0.10 nm and stabilizes near ~0.17–0.22 nm for the M34 binder. In **Figure S3**, the RMSD increases during the first ~1–1.5 ns and fluctuates around ~0.20–0.25 nm for the M97 binder.

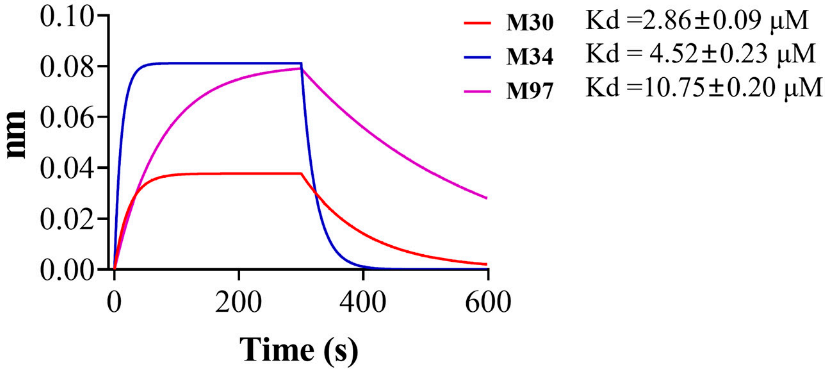

Figure S4

Figure S4 BLI sensorgrams for the binding of designed binders (M30, M34, M97) to the MMP9 PEX domain.Real-time binding responses (nm) are plotted against time (s). The calculated dissociation constants (KD) are: M30, 2.86±0.09 μM; M34, 4.52±0.23 μM; M97, 10.75±0.20 μM.

Supplementary Tables/Sequences

Table S1. Amino acid sequences of the de novo designed MMP9 binders.

Binder Name	Amino Acid Sequence
M30	PSVTVNIETIEQAELLNILIKEANLNIEELKELKDLETIKEKVKELEIKK
M34	MLLVRLTAEDPSKEKELLVEEERITEVIKEEFEKMVKEGIRSVSVEITEL
M97	VKVTEFLFPRRRLREVVEEARKLVEANPKVRVELEELDSGNVLARVIDEE

Note on Supplementary Files

Supplementary Data Files: The 3D atomic coordinates of the computationally designed MMP9 binder complexes are provided in PDB format as separate files within the accompanying .zip archive.
